# A deep learning approach to the diagnosis of atelectasis and attic retraction pocket in otitis media with effusion using otoscopic images

**DOI:** 10.1007/s00405-022-07632-z

**Published:** 2022-10-13

**Authors:** Junbo Zeng, Wenting Deng, Jingang Yu, Lichao Xiao, Suijun Chen, Xueyuan Zhang, Linqi Zeng, Donglang Chen, Peng Li, Yubin Chen, Hongzheng Zhang, Fan Shu, Minjian Wu, Yuejia Su, Yuanqing Li, Yuexin Cai, Yiqing Zheng

**Affiliations:** 1 Department of Otolaryngology, Sun Yat-Sen Memorial Hospital, Sun Yat-Sen University, 107# Yanjiang West Road, Guangzhou, China; 2 Shenshan Medical Center, Sun Yat-Sen Memorial Hospital, Sun Yat-Sen University, Shanwei, Guangdong China; 3 School of Automation Science and Engineering, South China University of Technology, Guangzhou, China; 4 Zhongshan School of Medicine, Sun Yat-Sen University, Guangzhou, China; 5 Department of Otolaryngology Head and Neck Surgery, The Third Affiliated Hospital of Sun Yat-Sen University, Guangzhou, China; 6 Department of Otolaryngology-Head and Neck Surgery, Zhujiang Hospital, South Medical University, Guangzhou, China

**Keywords:** Atelectasis, Attic retraction pocket, Otitis media with effusion, Deep learning

## Abstract

**Background:**

This study aimed to develop and validate a deep learning (DL) model to identify atelectasis and attic retraction pocket in cases of otitis media with effusion (OME) using multi-center otoscopic images.

**Method:**

A total of 6393 OME otoscopic images from three centers were used to develop and validate a DL model for detecting atelectasis and attic retraction pocket. A threefold random cross-validation procedure was adopted to divide the dataset into training validation sets on a patient level. A team of otologists was assigned to diagnose and characterize atelectasis and attic retraction pocket in otoscopic images. Receiver operating characteristic (ROC) curves, including area under the ROC curve (AUC), accuracy, sensitivity, and specificity were used to assess the performance of the DL model. Class Activation Mapping (CAM) illustrated the discriminative regions in the otoscopic images.

**Results:**

Among all OME otoscopic images, 3564 (55.74%) were identified with attic retraction pocket, and 2460 (38.48%) with atelectasis. The diagnostic DL model of attic retraction pocket and atelectasis achieved a threefold cross-validation accuracy of 89% and 79%, AUC of 0.89 and 0.87, a sensitivity of 0.93 and 0.71, and a specificity of 0.62 and 0.84, respectively. Larger and deeper cases of atelectasis and attic retraction pocket showed greater weight, based on the red color depicted in the heat map of CAM.

**Conclusion:**

The DL algorithm could be employed to identify atelectasis and attic retraction pocket in otoscopic images of OME, and as a tool to assist in the accurate diagnosis of OME.

## Introduction

Otitis media with effusion (OME) typically occurs due to persistent negative middle ear pressure and poor ventilation in the middle ear. Atelectasis and attic retraction pocket are the result of tympanic retraction in the pars tensa and pars flaccida, respectively, and nearly always occur concurrently with OME [1], although they could be the sequela of OME, especially since atelectasis is more frequently observed in cases of OME surgery [2, 3]. Atelectasis and attic retraction pocket are structural problems involving the tympanic membrane and include the possibility of chronic complaints and severity that progresses over time. Determining whether atelectasis or attic retraction pocket is present is an important feature of OME diagnosis [4]. Early diagnosis with appropriate follow-up enables practical methods for managing OME cases that include mild atelectasis or attic retraction pocket, and to promote natural self-healing [5, 6]. Severe atelectasis and attic retraction pocket nearly always requires additional surgery to treat the lesions [5, 7], as severe atelectasis and attic retraction pocket could predispose the affected individual to complications such as adhesive otitis, cholesteatoma formation, and erosion of the ossicles [8–12]. Many objective auxiliary diagnostic tools for OME are available, including tympanometry and pneumatic otoscopy [4]. However, the diagnosis of atelectasis and attic retraction pocket is typically based on otoscopy and assessment by an expert clinician. Several types of smartphone adaptable otoscopes can be used to acquire tympanic membrane images by either non-specialists or non-clinicians [13–16]. Diagnosis of ear disease made solely with manual examination and otoscopic images, however, has a low rate of accuracy, which may lead to an improper referral, delayed or improper treatment, and unnecessary follow-up. Previous research [17, 18] has found that the rate of correctly diagnosed otitis media by pediatricians was only 50% compared to that of 73% by otolaryngologists.

The progressive use of telemedicine and artificial intelligence in the otologic setting may gradually change the current approach to disease management. Previous studies have established machine learning models for the diagnosis of ear diseases that have achieved high diagnostic accuracy [19–27]. However, these studies regarded atelectasis and attic retraction pocket as one condition, rather than two distinct disorders. Moreover, OME and tympanic retraction were regarded as two separate diseases, overlooking the fact that these lesions can co-exist. When OME and tympanic retraction co-exist, standard diagnostic models diagnose only one lesion, such as OME [20, 21]. No DL studies have focused on dividing OME into different types according to the presence of atelectasis or attic retraction pocket.

In the present study, we developed and validated a DL model to identify the presence of attic retraction pocket and atelectasis in OME cases with the use of multi-center otoscopic images. We further classified OME into different types based on atelectasis and attic retraction pocket, which may be used to improve the procedures for accurate OME diagnosis and management.

## Material and methods

### Participant selection and acquisition of otoscopic images

OME otoscopic images from inpatients and outpatients were collected retrospectively from three hospitals between 2015 and 2019, including Sun Yat-sen Memorial Hospital of Sun Yat-sen University, the Third Affiliated Hospital of Sun Yat-sen University, and Zhujiang Hospital of Southern Medical University. Otoscopic images were taken with a 4-mm (KARL STORZ, Germany) or 2.7-mm (TIAN SONG, China) 0-degree otoscope by otolaryngologists. OME cases were confirmed with clinical criteria guidelines [4], including disease history, medical examination (otoscopic examination), and auditory tests (tympanometry). One to three best-quality otoscopic images from different angles with complete pars tensa and pars flaccida were obtained from each ear with at a resolution of at least 500 × 500 pixels. Otoscopic images with white light, and those that were neither overexposed nor underexposed were optimal. Otoscopic images with tympanostomy tubes, secretion, and earwax covering more than 20% of tympanic membranes were excluded in this study. This study was approved by the institutional review board of Sun Yat-Sen Memorial Hospital (reference number SYSKY-2022–130-01) and written informed consent was waved because of the retrospective nature of this study.

### Labeling of otoscopic images

Only a few OME otoscopic images depicting atelectasis and attic retraction pocket have been archived in electronic medical systems. To achieve a consistent process of identification, we did not assume that these records were necessarily correct. First, JBZ, with more than three years of otological experience, was assigned to classify the OME otoscopic images to determine whether there was evidence of atelectasis and attic retraction pocket, according to the first widespread standard for the assessment of atelectasis (retraction of the pars tensa severer than normal position) and attic retraction pocket (retraction in pars flaccida) [28, 29]. Because atelectasis and attic retraction pocket may appear in the same otoscopic image, these two lesions were identified step by step separately in the present study. This means when the identification of atelectasis was finished in the whole image dataset, the identification of attic retraction pocket was just started. Subsequently, two otologists (XYZ and WTD), with more than 10 years of clinical experience in otology, were assigned to independently review the identified lesions, and any discrepancies would be discussed with a third otologist (YXC) with more than 10 years of clinical experience in otology, until a consensus was reached. Similar to actual clinical practice, the prevalence of the different stages of atelectasis and attic retraction pocket was heavily skewed in our dataset, with stage III and IV atelectasis and attic retraction pocket occurring at a rate of less than 5%. To ensure that sufficient otoscopic images were available to develop and assess the performance of this model, we considered cases of atelectasis and attic retraction pocket without stage classification. Other clinical demographic data, such as audiology test results, age, and gender, were not used to develop the DL model.

### DL model development and validation

A threefold random cross-validation based on patients was adopted to divide the dataset into training and validation sets, as proposed in a previous study [19]. The output from this model was derived from two standard two-class tasks (task 1: determining whether attic retraction pocket is present in the OME otoscopic images, and task 2: whether atelectasis is present in the OME otoscopic images), both of which were developed separately. All otoscopic images were converted into 299 × 299 pixels as input data. We used a convolutional neural network (CNN) model (Inception-V3) pre-trained on the ImageNet dataset (http://www.image-net.org). Otoscopic images from this dataset were subsequently used to fine-tune the hyperparameters of the pre-trained CNN model. Stochastic gradient descent optimization was used with the following parameter settings: batch size = 18, initial learning rate = 0.001, decay = 0.1, momentum = 0.9, epsilon = 1e-10. During the training process, online data were used for data expansion, including random vertical and horizontal flip, and constant aspect ratio scaling. The DL model consisted of a CNN that implicitly recognized characteristics of attic retraction pocket and atelectasis from otoscopic images. To evaluate the performance of the CNN model in clinical practice, we compared the predicted diagnosis with the standard diagnostic label using the threefold average classification of accuracy, sensitivity, and specificity of the model (normal pars flaccida vs. attic retraction pocket and normal pars tensa vs. atelectasis). We also used the receiver operating characteristic (ROC) curve and area under the ROC curve (AUC) to determine the diagnostic capability of the DL model in identifying the presence of atelectasis and attic retraction pocket in OME otoscopic images.

### Class activation mapping

Class Activation Mapping (CAM) [30] was employed to visualize the discriminative region of the CNN model in the OME otoscopic images. CAM used different colors to show various values of the DL model, ranging from blue (non-specific region) to red (most discriminative region), which is a common method used in otologic research with artificial intelligence [20, 23, 24]. The correct identification of the lesion area, depicted in red in the otoscopic images, is essential for clinicians to have confidence in the DL model. All experiments were operated with Python (version 3.6) in Keras (version 2.2.4) using the Python programming language. The diagnostic model was developed based on TensorFlow and carried out with 4 Titan XP 256 GB GPU.

## Results

We collected an image dataset consisting of 6393 OME otoscopic images of which 3564 (55.74%) OME otoscopic images were identified with attic retraction pocket, and 2829 (44.26%) with normal pars flaccida. In addition, atelectasis was diagnosed in 2460 (38.48%) OME otoscopic images, and 3933 (61.52%) were identified with normal pars tensa. Each otoscopic image was reviewed by at least three expert otologists.

### DL model performance

**Fig. 1:**
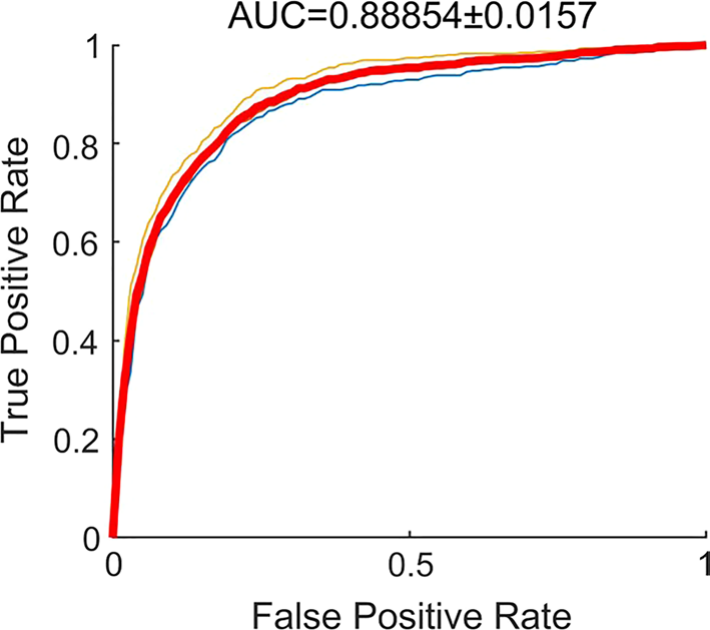
Receiver–operator characteristic (ROC) curves and corresponding area under the ROC curve (AUC) of the deep learning model for the detection of attic retraction pocket

**Fig. 2:**
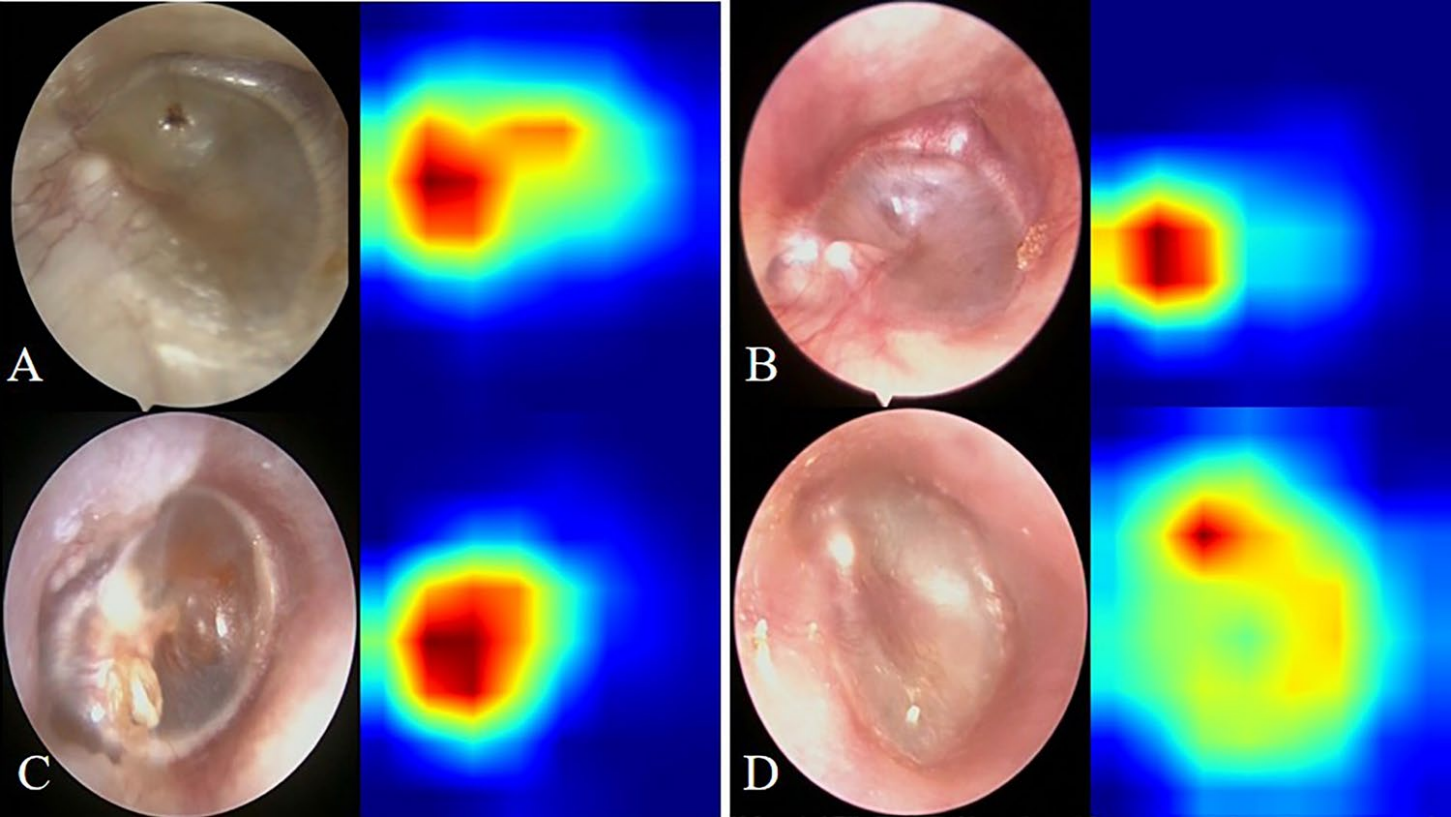
Class activation maps of the deep learning model for the detection attic retraction pocket. Class activation maps of the identification of attic retraction pockets. The red-colored area represents the discriminative region in the otoscopic images, whereas the blue-colored area represents the non-specific region in the otoscopic images. **A** Normal pars flaccida. **B**–**D** Attic retraction pocket

We used threefold cross-validation for developing and testing the CNN model to detect OME referable attic retraction pocket and atelectasis. The AUC of classifying the normal pars flaccida and attic retraction pocket was 0.89, and the average accuracy, sensitivity, and specificity were 89%, 0.93, and 0.62, respectively (Fig. 1). The CNN model achieved an AUC of 0.87 in classifying the normal pars tensa and atelectasis, and the average accuracy, sensitivity, and specificity were 79%, 0.71, and 0.84 respectively (Fig. 2).

### Class activation map

**Fig. 3:**
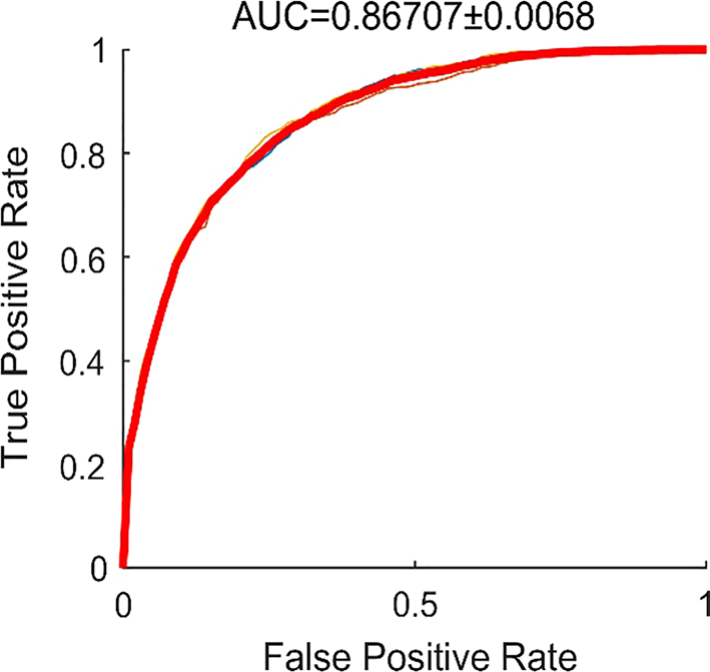
Receiver–operator characteristic (ROC) curves and corresponding area under the ROC curve (AUC) of the deep learning model for the detection of atelectasis

**Fig. 4:**
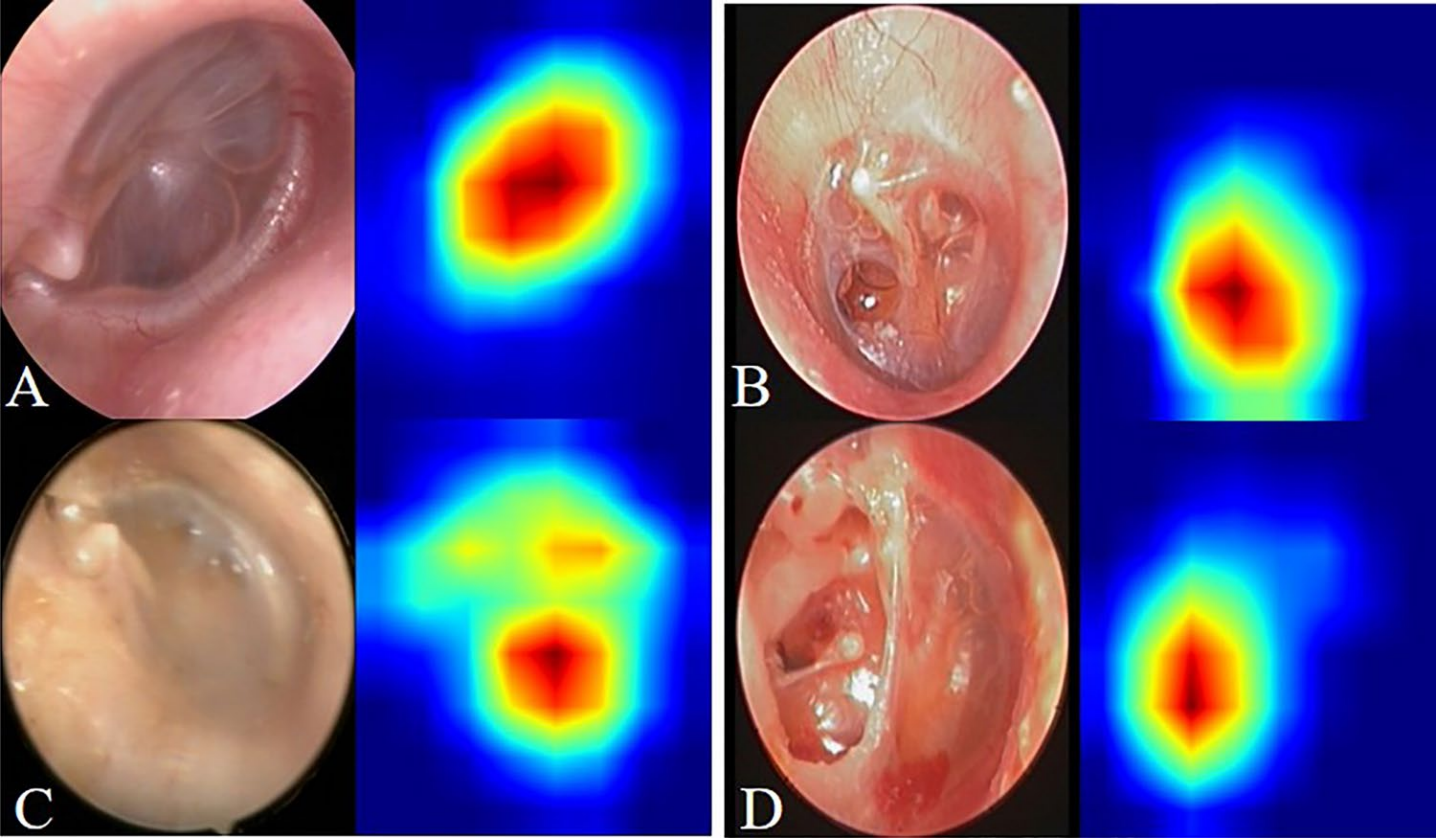
Class activation maps of the deep learning model for the detection of atelectasis. Class activation maps of the deep learning model for the detection of atelectasis. The red-colored area represents the discriminative region in the otoscopic images, whereas the blue-colored area represents a non-specific region in the otoscopic images. **A** Normal pars tensa. **B**–**D** Atelectasis and attic retraction pocket

The heat map of the CAM image was generated using otoscopic images from the validation set. The CAM showed that the DL model was capable of identifying attic retraction pocket accurately with the color red, with deeper or larger cases of attic retraction pocket possessing more values (Fig. 3). In addition, partial atelectasis and general atelectasis were identified by the DL model, and deeper or larger atelectasis showed more values in red (Fig. 4).

## Discussion

In this study, we developed and validated a DL model to get an accurate diagnosis of OME by identifying the presence of attic retraction pocket and atelectasis with multiple centers of OME otoscopic images. The DL model could be used to determine further classification of OME by attic retraction pocket and atelectasis. This CNN algorithm obtained an AUC of 0.89 for the identification of attic retraction pocket and 0.87 for atelectasis in OME otoscopic images. The CAM of the DL model showed a consistent discriminative region of tympanic membranes to otolaryngologists.

Attic retraction pocket and atelectasis are the most commonly observed tympanic changes in OME, which currently lacks an auxiliary diagnostic tool. Different types of otoscopes (e.g., smartphone-based imaging otoscopes [13, 14, 16]) are the preferred diagnostic modality for diagnosing attic retraction pocket and atelectasis, but these approaches are limited for use by clinicians who lack sufficient diagnostic experience, particularly in clinicians who are not specialists in otolaryngology. During the procedures carried out for OME management, attic retraction pocket and atelectasis could be detected by our DL model. In clinical practice, the DL model could be useful in diagnosing attic retraction pocket and atelectasis and for assisting clinicians in determining a more accurate diagnosis. In addition, the DL diagnostic model could increase the performance of clinicians in diagnosing these disorders. Then, precise diagnosis with DL model could save the economic cost of misdiagnosis and missed diagnosis. Including avoiding unnecessary referral and surgery, and facilitating the screening of severe OME cases. Besides, DL diagnostic model could be loaded on the website, and could be used by patients, while tympanometry and otoscope always need additional professionals, which will increase the cost of disease diagnosis.

When the attic retraction pocket or atelectasis occurs in OME cases, patients should be immediately referred to otolaryngologists for further evaluation. Moreover, for young otolaryngologists and those not specialized in otolaryngology, this model could be used as a study tool to increase their knowledge of attic retraction pocket and atelectasis.

Previous studies have established DL models for the diagnosis of tympanic retraction and achieved an average level of accuracy ranging from 85.78 to 88.06% [20, 22]. However, these previous studies regarded tympanic retraction and OME as different conditions, which ignores those cases in which tympanic retraction and OME are both potentially present. Cha et al. [20] proposed a DL model that detected attic retraction pocket or adhesive otitis media with an accuracy of 85.78%. However, these authors merged atelectasis and attic retraction pocket into a single class rather than distinguish them as two different conditions. Moreover, a disease labeling approach was used when there was more than one feature in otoscopic images, in which only the more severe feature was identified. For example, when OME and attic retraction pocket were both present, the DL model only provided an output for the diagnosis of OME. As a result, such an approach will result in disregarding cases of milder disease. Shie et al. [21] extracted color, geometric, and textural features to develop a classification system for differentiating most types of otitis media, achieving an accuracy of 88.06%; however, the machine learning model was developed in a small dataset that included 865 otoscopic images. Moreover, the color of otoscopic images may vary with diverse conditions for illumination and different otoscope systems; thus, color is not a stable variable in an accurate diagnostic model. During the course of disease, attic retraction pocket is likely to progress to cholesteatoma, and atelectasis may evolve to include ossicular erosions [31]. In addition, the surgical approach to severe tympanic membranes in pars tensa and pars flaccida widely differs. Therefore, we divided tympanic retraction into atelectasis and attic retraction pocket. Compared to previous models, we were able to detect the atelectasis and attic retraction pocket separately. This image classification system, therefore, was the first to individually diagnose two types of tympanic membrane lesions. The performance of the DL model in the present study has been found to be better than the diagnostic performance of pediatricians, general practitioners, and otolaryngologists, with rates of accuracy ranging from 50 to 80% [17, 32, 33]. This DL model could provide an objective second opinion to assist otolaryngologists in making a correct diagnosis. The performance of this DL model is comparable to the diagnostic accuracy of tympanometry in diagnosing OME, with a degree of sensitivity ranging from 76 to 96% [34, 35].

Our results showed that DL models could identify different regions (pars tensa and pars flaccida) of retraction on the tympanic membrane with varying degrees of performance. Based on clinical experience, it is reasonable to suggest that attic retraction pocket is easier to identify than atelectasis because the normal tympanic membrane shows a mild retraction in the pars tensa without a retraction in the pars flaccida. Thus, in cases of mild retraction, atelectasis may be subtle and difficult to determine, whether it is normal or at stage I atelectasis. Our image dataset was representative and collected from three hospitals with different types of otoscopes, photo conditions, and record systems.

Considering our experience [19] and that of other teams [20, 24, 26, 27], Google Inception-V3 demonstrated high performance in developing a diagnostic DL model based on otoscopic images. Therefore, the Google Inception-V3 CNN model was adopted as the backbone network, and subsequently trained, tuned, and evaluated. Based on our findings, the foremost approach to OME otoscopic images with atelectasis by CAM was to focus on pars tensa and attic retraction pocket by CAM on pars flaccida, particularly large and deep retraction pockets and atelectasis, which is consistent with current practice by otologists.

### Limitations

Some limitations in our study should be noted. Although the CNN algorithm could identify mild and severe attic retraction pocket and atelectasis, due to the low incidence of severe attic retraction pocket and atelectasis [36–38], however, there were not enough images to develop and validate a DL model to identify the different stages of attic retraction pocket and atelectasis. This limitation meant that clinicians were required to complete the task of further classification. Moreover, the retrospective nature of the design created some limitations with regard to data collection, such as inconsistent illumination of the otoscopes. The accuracy of the DL model was considerable affected by the quality of otoscopic images, which is associated with the operators’ examination skills and with the cooperation of the children being examined. Larger, prospective studies with more detailed data for collection rules are needed to improve the performance of the DL model. Finally, non-medical history and hearing information were provided for the DL model and to the otolaryngologists, which may have affected the accuracy of diagnosis. Clinicians could improve the accuracy of diagnosis by combining information from disease history.

## Conclusion

In summary, we developed and validated a deep learning model using otoscopic images to diagnose attic retraction pocket and atelectasis in patients with OME, which could be useful in assisting junior otolaryngologists and non-otolaryngologists when making the appropriate diagnosis.

## Data Availability

The datasets used and analysed during the current study are available from the corresponding author on reasonable request.

## References

[1] Ungkanont K, Charuluxananan S, Komoltri C (2010). Association of otoscopic findings and hearing level in pediatric patients with otitis media with effusion. Int J Pediatr Otorhinolaryngol.

[2] De Beer BA, Schilder AG, Zielhuis GA, Graamans K (2005). Natural course of tympanic membrane pathology related to otitis media and ventilation tubes between ages 8 and 18 years. Otol Neurotol.

[3] Stenstrom R, Pless IB, Bernard P (2005). Hearing thresholds and tympanic membrane sequelae in children managed medically or surgically for otitis media with effusion. Arch Pediatr Adolesc Med.

[4] Rosenfeld RM, Shin JJ, Schwartz SR, Coggins R, Gagnon L, Hackell JM (2016). Clinical practice guideline: otitis media with effusion executive summary (update). Otolaryngol Head Neck Surg.

[5] Spielmann P, Mills R (2006). Surgical management of retraction pockets of the pars tensa with cartilage and perichondrial grafts. J Laryngol Otol.

[6] Garcia de Hombre AM (2005). Bibliographic revision of retraction pockets handling in relation to surgical treatment. An Otorrinolaringol Ibero Am.

[7] Saunders JE (2008). Does early surgical intervention of middle ear atelectasis improve long-term results and prevent cholesteatoma?. Arch Otolaryngol Head Neck Surg.

[8] Lee JH, Hong SM, Kim CW, Park YH, Baek SH (2015). Attic cholesteatoma with tiny retraction of pars flaccida. Auris Nasus Larynx.

[9] Ostrowski VB, Bojrab DI (2003). Minimally invasive laser contraction myringoplasty for tympanic membrane atelectasis. Otolaryngol Head Neck Surg.

[10] Cutajar J, Nowghani M, Tulsidas-Mahtani B, Hamilton J (2018). The natural history of asymptomatic deep pars tensa retraction. J Int Adv Otol.

[11] Kim GW, Jung HK, Sung JM, Kim JS, Kim CW (2020). A tiny retraction of the pars flaccida may conceal an attic cholesteatoma. Eur Arch Otorhinolaryngol.

[12] Alzahrani M, Saliba I (2014). Tympanic membrane retraction pocket staging: is it worthwhile?. Eur Arch Otorhinolaryngol.

[13] Samra S, Wu A, Redleaf M (2016). Interactive iPhone/iPad App for increased tympanic membrane familiarity. Ann Otol Rhinol Laryngol.

[14] Ni G, Curtis S, Kaplon A, Gildener-Leapman N, Brodsky J, Aaron K (2021). Development of video otoscopy quiz using a smartphone adaptable otoscope. J Otol.

[15] Myburgh HC, van Zijl WH, Swanepoel D, Hellstrom S, Laurent C (2016). Otitis media diagnosis for developing countries using tympanic membrane image-analysis. EBioMedicine.

[16] Cavalcanti TC, Kim S, Lee K, Lee SY, Park MK, Hwang JY (2020). Smartphone-based spectral imaging otoscope: system development and preliminary study for evaluation of its potential as a mobile diagnostic tool. J Biophotonics.

[17] Pichichero ME, Poole MD (2001). Assessing diagnostic accuracy and tympanocentesis skills in the management of otitis media. Arch Pediatr Adolesc Med.

[18] Pichichero ME, Poole MD (2005). Comparison of performance by otolaryngologists, pediatricians, and general practioners on an otoendoscopic diagnostic video examination. Int J Pediatr Otorhinolaryngol.

[19] Cai Y, Yu JG, Chen Y, Liu C, Xiao L, Grais EM (2021). Investigating the use of a two-stage attention-aware convolutional neural network for the automated diagnosis of otitis media from tympanic membrane images: a prediction model development and validation study. BMJ Open.

[20] Cha D, Pae C, Seong SB, Choi JY, Park HJ (2019). Automated diagnosis of ear disease using ensemble deep learning with a big otoendoscopy image database. EBioMedicine.

[21] Shie CK, Chang HT, Fan FC, Chen CJ, Fang TY, Wang PC (2014). A hybrid feature-based segmentation and classification system for the computer aided self-diagnosis of otitis media. Annu Int Conf IEEE Eng Med Biol Soc.

[22] Wu Z, Lin Z, Li L, Pan H, Chen G, Fu Y (2021). Deep learning for classification of pediatric otitis media. Laryngoscope.

[23] Lee JY, Choi S-H, Chung JW (2019). Automated classification of the tympanic membrane using a convolutional neural network. Appl Sci.

[24] Khan MA, Kwon S, Choo J, Hong SM, Kang SH, Park IH (2020). Automatic detection of tympanic membrane and middle ear infection from oto-endoscopic images via convolutional neural networks. Neural Netw.

[25] Cavalcanti TC, Lew HM, Lee K, Lee SY, Park MK, Hwang JY (2021). Intelligent smartphone-based multimode imaging otoscope for the mobile diagnosis of otitis media. Biomed Opt Express.

[26] Sundgaard JV, Harte J, Bray P, Laugesen S, Kamide Y, Tanaka C (2021). Deep metric learning for otitis media classification. Med Image Anal.

[27] Senaras C, Moberly AC, Teknos T, Essig G, Elmaraghy C, Taj-Schaal N (2018) Detection of eardrum abnormalities using ensemble deep learning approaches. In: Medical Imaging 2018: Computer-Aided Diagnosis. SPIE

[28] Sade J, Berco E (1976). Atelectasis and secretory otitis media. Ann Otol Rhinol Laryngol.

[29] Tos M, Stangerup SE, Larsen P (1987). Dynamics of eardrum changes following secretory otitis. A prospective study. Arch Otolaryngol Head Neck Surg.

[30] Selvaraju RR, Cogswell M, Das A, Vedantam R, Parikh D, Batra D (2017) Grad-CAM: Visual Explanations from Deep Networks via Gradient-Based Localization. In: 2017 IEEE International Conference on Computer Vision (ICCV). IEEE

[31] Alper C, Olszewska E (2017). Assessment and management of retraction pockets. Otolaryngol Pol.

[32] Buchanan CM, Pothier DD (2008). Recognition of paediatric otopathology by General Practitioners. Int J Pediatr Otorhinolaryngol.

[33] Kuruvilla A, Shaikh N, Hoberman A, Kovacevic J (2013). Automated diagnosis of otitis media: vocabulary and grammar. Int J Biomed Imaging.

[34] Takata GS, Chan LS, Morphew T, Mangione-Smith R, Morton SC, Shekelle P (2003). Evidence assessment of the accuracy of methods of diagnosing middle ear effusion in children with otitis media with effusion. Pediatrics.

[35] Muderris T, Yazici A, Bercin S, Yalciner G, Sevil E, Kiris M (2013). Consumer acoustic reflectometry: accuracy in diagnosis of otitis media with effusion in children. Int J Pediatr Otorhinolaryngol.

[36] Cassano M, Cassano P (2010). Retraction pockets of pars tensa in pediatric patients: clinical evolution and treatment. Int J Pediatr Otorhinolaryngol.

[37] Tos M, Poulsen G (1980). Attic retractions following secretory otitis. Acta Otolaryngol.

[38] Sade J, Fuchs C, Luntz M (1997). Shrapnell membrane and mastoid pneumatization. Arch Otolaryngol Head Neck Surg.

